# Impact of Tire-Derived Microplastics on Microbiological Activity of Aerobic Granular Sludge

**DOI:** 10.3390/ijms26094136

**Published:** 2025-04-27

**Authors:** Weronika Irena Mądzielewska, Piotr Jachimowicz, Job Oliver Otieno, Agnieszka Cydzik-Kwiatkowska

**Affiliations:** 1 Department of Environmental Biotechnology, University of Warmia and Mazury in Olsztyn, Słoneczna 45G, 10-709 Olsztyn, Poland; job.otieno@student.uwm.edu.pl; 2 Institute of Environmental Technology, Centre for Energy and Environmental Technologies, VSB-Technical University of Ostrava, 708 00 Ostrava, Czech Republic; piotr.jachimowicz@vsb.cz

**Keywords:** tire wear particles, aerobic granular sludge, batch reactor, *amoA*, *nirK*, *nosZ*

## Abstract

In recent years, there has been an increase in the emission of tire wear particle (TWP) microplastics from wastewater treatment plants into the environment. The aim of this study was to determine the effect of TWPs in wastewater flowing into a biological reactor on the transcription of the *16S rRNA* gene and the key genes responsible for nitrogen metabolism, *amoA*, *nirK* and *nosZ*, in aerobic granular sludge. The laboratory experiment was carried out in sequencing aerobic granular sludge reactors operated in an 8 h cycle into which TWP microplastics were introduced with municipal wastewater at a dose of 50–500 mg TWPs/L. The ammonia removal rate and the production of oxidized forms of nitrogen increased with the TWP dose. Gene transcript abundance analysis showed that the presence of rubber and substances leached from it promoted the activity of ammonium-oxidizing bacteria (160% increase), while the transcription of genes related to denitrification conversions was negatively affected. The activity of nitrite reductase gradually decreased with increasing TWP concentration in wastewater (decreased by 33% at 500 mg TWPs/L), while nitric oxide reductase activity was significantly inhibited even at the lowest TWP dose (decreased by 58% at 500 mg TWPs/L). The data obtained indicate that further studies are needed on the mechanisms of the effects of TWPs on the activities of the most important groups of microorganisms in wastewater treatment to minimize the negative effects of TWPs on biological wastewater treatment.

## 1. Introduction

In 2023, global plastic production exceeded 413 million tons [1], with Europe contributing 54 million tons. This phenomenon, which is due to inadequate waste management [2], is accompanied by an increase in plastic waste and its degradation into fine particles. Microplastic (MP) is a plastic with a diameter of less than 5 mm [3]. Common polymer types are polyethylene (PE), polypropylene (PP), polystyrene (PS) and polyester (PES) [4]. There are two main sources of MP: primary MPs, which are produced by the release of small particles during industrial processes, and secondary MPs, which are detached from larger fragments of plastics [5]. Plastic pellets, personal care products, laundry wastewater, municipal waste, artificial turf and abrasion from car tires are considered to be the most important sources of MP [6].

According to a study by Baensch-Baltruschat et al. [7], tire and road surface particles generated by the abrasion of tire materials on asphalt are one of the main sources of MP emissions into the environment, taking into account the recommendations of the classification of plastic pollutants considering elastomers (synthetic rubbers) and highly modified natural polymers (vulcanized natural rubber) [8]. According to Eurostat (2022) [9], the average number of passenger cars in Europe is 560 per 1000 inhabitants, and the global estimated emission of MPs from tire wear is 5.9 million tons per year, equivalent to 0.81 kg per person annually [9]. Higher traffic intensity leads to an increase in the concentration of microplastics (MPs) and tire wear particles (TWPs) in road snow. In a study by Mierzyńska et al. [10], the concentration of MPs in Suwałki, Poland, ranged from 62.32 particles/L on roads with low traffic intensity to 792.76 particles/L on roads with heavy traffic intensity. Tires mainly contain natural rubber, synthetic rubber, carbon black, steel wire for belts, polyester and nylon fibers, steel wire for the casing, as well as other chemical substances such as waxes, oils, dyes, silica, and clays; rubber and elastomers account for about 46% of the tire mass [11].

Due to their size, MP particles easily flow into the sewer system and end up in wastewater treatment plants (WWTPs). In stormwater runoff in Australia, for example, the average concentration of MP in wastewater was 14.2 MPs/L [12]. More than 30 types of MP have been detected in WWTP influents and effluents, including PES, PE, polyethylene terephthalate (PET) and polyamide (PA). They occur in concentrations of 1 to 10,044 MPs/L in the influent and 0 to 477 MPs/L in the effluent [13,14]. The average annual MP emissions from WWTPs may reach 2 million particles/day, despite a treatment efficiency of more than 97% [13]. MP is mainly present in the form of fibers [15] and is a high-risk source of pollution given MP’s tendency to sorb pollutants such as polycyclic aromatic hydrocarbons, polychlorinated biphenyls and heavy metals [16].

Aerobic granular sludge (AGS) technology is increasingly used in wastewater treatment systems due to its high efficiency of carbon, phosphorus and nitrogen compounds removal [17], rapid separation of biomass from treated wastewater [18] and high microbial biodiversity that promotes degradation of micropollutants such as, e.g., bisphenol A (BPA) and antibiotics [19,20,21].

The abundance of microorganisms in the AGS multilayer structure promotes the co-metabolic consumption of produced substrates [22], which has a positive effect on the intensity of biological wastewater treatment processes. Co-metabolism has repeatedly been identified as an important mechanism for the removal of micropollutants [23]. Alternating high and low concentrations of organic matter in the operating cycle of aerobic granular reactors promote increased polymer storage by bacteria, which facilitates the competition of nitrifiers with heterotrophs on the granule surface [24].

A high concentration of MPs negatively affects nitrification. Li et al. [25] observed that at MP concentrations (mainly PP) above 5000 particles/L, nitrification was disrupted, and ammonia accumulation occurred due to a reduction in the ammonia oxidation rate, directly proportional to the amount of MP. In addition, MP caused an inhibition of nitrite oxidation. In the case of denitrification, it was shown that polyvinyl chloride (PVC) in an amount of 1000 to 10,000 particles/L could interfere with denitrification by reducing its rate, while other MPs (PE, PS, PES) in an amount of more than 5000 particles/L improved the intensity of denitrification.

MP from car tire wear particles (TWPs) and other types of MP have many similarities, including chemical stability, and resistance to degradation or hydrophobicity [26]. Most MP is removed from WWTPs as a result of retention in biomass. For TWPs, they can also be the main removal mechanism, although it is difficult to retain TWPs smaller than 63 µm in sludge, and their treatment efficiency can drop to as low as 50% [27].

The efficiency of wastewater treatment depends not only on the abundance of bacteria in the environment, but more importantly on their activity. A large number of nitrifying bacteria (e.g., *Nitrosomonas* sp.) and denitrifying bacteria (e.g., *Thauera* sp.) does not guarantee high efficiency of wastewater treatment process if their enzymatic activity is low [28]. To determine how selected compounds present in wastewater affect the wastewater treatment process, especially the most important processes such as nitrification and denitrification, molecular methods can be used. The most common markers for nitrification and denitrification processes are the genes coding for ammonium monooxygenase (*amoA*), nitrite reductase (*nirK*, *nirS*) and nitric oxide reductase (*nosZ*).

Despite the growing number of studies on the presence of MP in various aquatic environments, such as sediments of highway runoff, river runoff and lakes [29], the detailed molecular analysis of the effects of MP, especially at the level of gene expression, remains an under-researched area. In the case of TWPs, the disruption of the efficiency of biological processes may result not only from their physical presence and their effects on the structure of the biomass, but also from the release of toxic compounds that are a component of the tire material. Therefore, the present study investigated the nitrogen removal kinetics and expression of genes critical for nitrogen metabolism (*amoA*, *nirK*, *nosZ*) and the overall activity of the microbiome (*16S rRNA*) in aerobic granules exposed to increasing doses of TWP in wastewater. By uniquely bridging kinetic and molecular analyses, this study offers a comprehensive understanding of how TWPs influence key microbial processes in wastewater treatment, contributing valuable knowledge for optimizing the stability and efficiency of GSBR systems.

## 2. Results and Discussion

### 2.1. Impact of TWP on Nitrogen Compound Transformations in GSBRs

**Figure 1:**
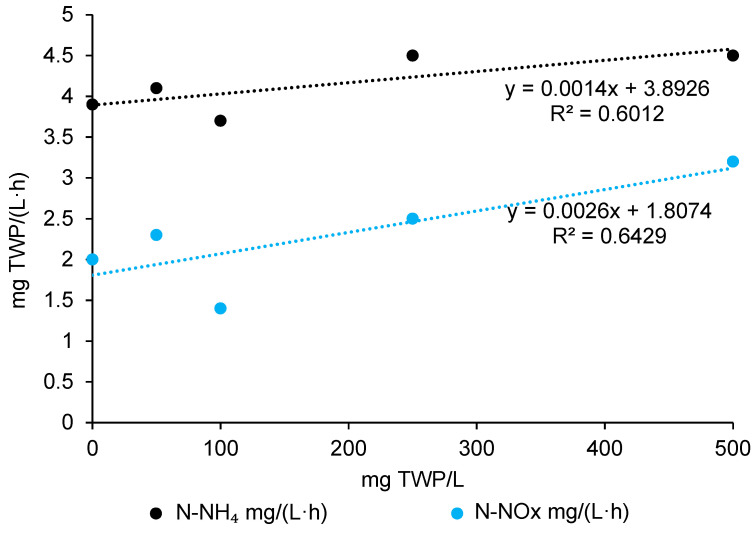
Impact of TWP on ammonium and nitrogen removal kinetics in GSBR cycle.

The presence of TWP affected the kinetics of pollutant transformations in the GSBR cycle. The rate of ammonium removal increased with increasing TWP concentrations in the wastewater from 3.9 mg N-NH_4_/(L·h) in the control reactor to 4.5 mg N-NH_4_/(L·h) in the TWP_500 (Figure 1, Table A2). Depending on the type of wastewater and reactor operation, the rate of ammonium nitrogen removal by granular sludge may vary from 1.25 mg N-NH_4_/(L·h) to 598 mg N-NH_4_/(L·h) [30,31,32,33]. In a study by Jachimowicz et al. [34] in the series where 10–50 mg MP/L such as PE and PET were dosed, it was observed that the removal rate of N-NH_4_ was higher than in the control reactor. MP can to rapidly sorb N-NH_4_ ions on its surface, leading to an increase in the volumetric ammonium removal rate of AGS. The substances released from MP present in the reactor may serve as an energy source, further enhancing the process. Additionally, MP, by inducing oxidative stress, may promote the release of reactive oxygen species, which positively influences nitrification [15,35].

The NOx concentrations in the GSBR effluents showed a dose-dependent relationship with TWP—increasing TWP concentrations favored a faster production of oxidized nitrogen forms (NOx). Additionally, a significant correlation between N-NH_4_ and N-NOx (r = 0.91) indicates a strong link between ammonium oxidation and NOx accumulation in the system. At 500 mg TWP/L wastewater, NOx production increased to 3.2 mg N-NOx/(L·h), which was 60% higher than in the control reactor. The TWP fraction of 70–200 μm may disrupt membrane transport in bacterial cells, while TWP accumulation inside the granules could potentially affect substrate transport between different oxic within the AGS. Disruption of the membrane transport or substrate penetration could disturb the microbial processes ultimately affecting the functionality and stability of the AGS. However, this does not appear to result in a significant reduction in the kinetics of the nitrogen oxide removal [36].

### 2.2. Microbial Activity

In our study, gene expression was determined by real-time PCR at specific time points during an 8 h GSBR cycle, which included the anaerobic, anoxic and aerobic phases. Each of these phases places different metabolic demands on the microbial community, leading to coordinated changes in gene expression over time. Additionally, the addition of a carbon source at the beginning of the GSBR cycle must be taken into account.

**Figure 2:**
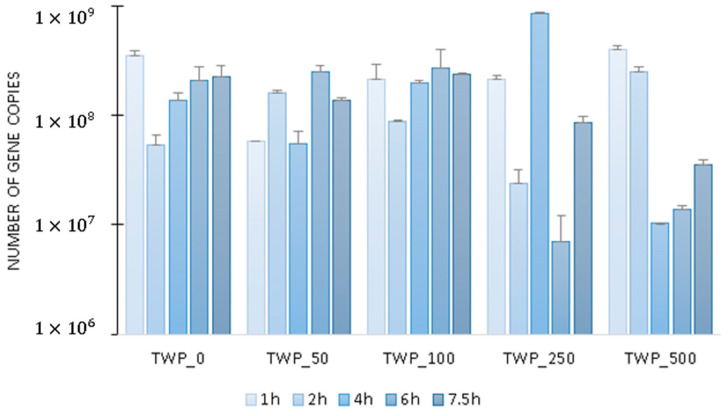
The number of copies of *16S rRNA* gene transcripts in the GSBR cycle.

The effect of TWP on total microbiome activity was determined based on *16S rRNA* gene expression. It was found that in the control reactor and the reactors operated with 2 lower doses of TWP, the level of *16S rRNA* was similar, with increasing values at 6 and 7 h of the cycle (Figure 2). In the reactors operated with the two highest TWP doses, there was a significant decrease in microbial activity in the middle of the cycle: in TWP_500, the *16S rRNA* copy number decreased from 399 ± 39 million transcripts at 1 h of the cycle to 10 ± 1 million transcripts at 4 h of GSBR operation. At the end of the cycle, activity slowly increased again. It can be assumed that the compounds released from TWP inhibited microbial activity, and that most microorganisms regained their activity after the metabolism of the substrates released from TWP. Examples of activity-inhibiting compounds that can be released from TWP are BPA, heavy metals, polycyclic aromatic hydrocarbons and aniline [34,37,38]. Additionally, it was determined that the largest divergence in the *16S rRNA* gene copy number at different hours of the GSBR cycle occurred in the TWP_250 reactor.

A significant positive correlation (r = 0.49) was found between the TWP dose in wastewater and the average activity of nitrifying bacteria, expressed by the number of *amoA* transcripts during the GSBR operating cycle (Table A3).

**Figure 3:**
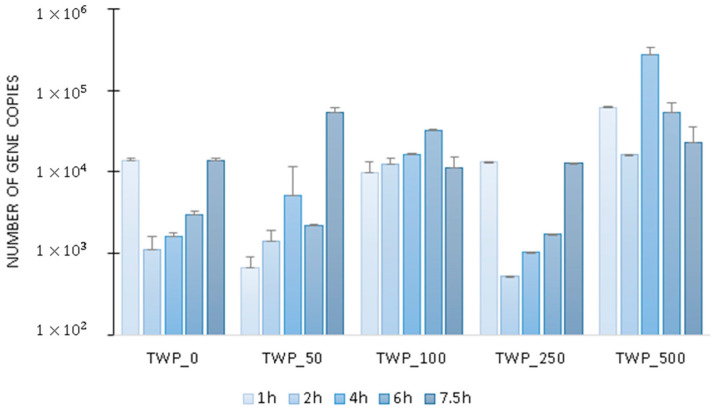
The number of copies of the *amoA* gene transcripts in the GSBR cycle.

The number of *amoA* transcripts (Figure 3) tended to increase during the aeration phase in the control reactor and in the reactors operated with a TWP dose in the effluent of 250 mg/L or less. The copy number of *amoA* gene transcripts varied from 519 ± 0 at 2 h of the TWP_250 cycle to 52,760 ± 9481 at the end of the operational cycle in TWP_50. It can be concluded that, as the concentration of TWP in the system increased, nitrogen co-metabolism occurred; i.e., aromatic tire additives containing nitrogen, such as aniline, were degraded as an alternative energy source [39,40], resulting in stimulation of ammonium monooxygenase activity. This is particularly beneficial under conditions of organic carbon deficiency. A similar phenomenon was observed in the study by Cydzik-Kwiatkowska et al. [37], where addition of 12 mg BPA/L in synthetic municipal wastewater contributed to a 1.5-fold increase in the N-NH_4_ removal rate.

The highest number of *amoA* transcripts was recorded at 4 h of the operational cycle in TWP_500 reaching 275,000 ± 62,000 copies. It can therefore be assumed that in this GSBR, the highest number of N-containing compounds, including those eluted from TWP, increased nitrification intensity [41].

An analysis of genes related to the denitrification process was also carried out, indicating that TWP negatively affected denitrification activity. Intensity of denitrification was affected by availability of organic carbon compounds and nitrification products as well as oxic conditions in the biomass [42]. Gradual depletion of organics in the cycle as well as incorporation of TWP in granule structure worsened sludge stability and decreased anoxic zones in the granule structure [43]. This was confirmed by a significant negative correlation between the expression levels of the nitrite reductase gene *nirK* (r = −0.61) and the nitric oxide reductase gene *nosZ* (r = −0.47) and the GSBR cycle time. According to the study by Li et al. [25], an increase in denitrification intensity in the Wuhan municipal wastewater treatment plant during the reactor cycle was only observed at a dose of ≥10,000 MP/L (PVC, PE, PS). The uneven MP surface was a settlement site for nitrogen oxide-reducing bacteria supporting their effective growth in reactors.

**Figure 4:**
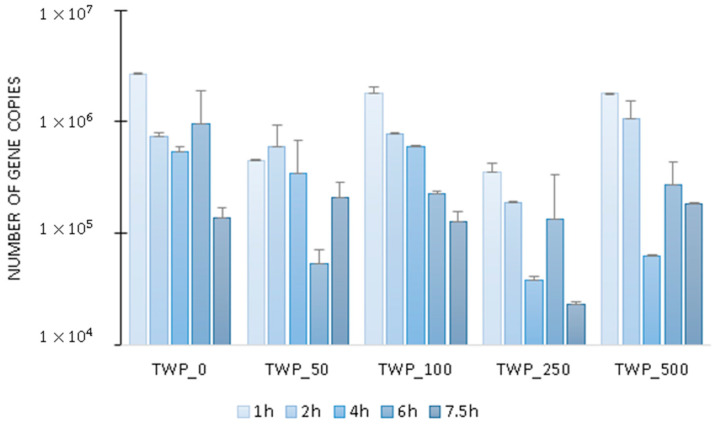
The number of copies of the *nirK* gene transcripts in the GSBR cycle.

In the study, the difference in the activity of denitrifying microorganisms per cycle (Figure 4) ranged from 331,000 transcripts for TWP_250 (the minimum was recorded at 7.5 h of the cycle and the maximum at 1 h of the cycle) to 3 million copies of the *nirK* gene for TWP_0 (the minimum was recorded at 7.5 h of the cycle and the maximum at 1 h of the cycle). The copy number of the *nirK* gene ranged from 23,000 ± 2000 at 7.5 h of the cycle (TWP_250) to 3 ± 0.04 million at 1 h of the cycle (TWP_0).

A significant positive correlation (r = 0.74) was found between the copy number of the *nirK* gene and the copy number of the *nosZ* gene. This indicates that the expression of the *nosZ* gene was co-regulated by the activity of the *nirK* gene [44], which is crucial for complete denitrification. Complete denitrification enables reducing greenhouse gas emissions. The addition of PE and polylactic acid can lead to an increase in N_2_O emissions by, respectively, 15.96% and 21.52% in acidified tobacco soils over an incubation period of 35 days [45]. Consequently, a lower activity of denitrifiers that reduce nitric oxide may contribute to ozone depletion by excessive N_2_O emission during wastewater treatment [46]. However, there are few studies addressing the effects of MPs and even fewer on TWPs and their effect on the efficiency of biological wastewater treatment.

**Figure 5:**
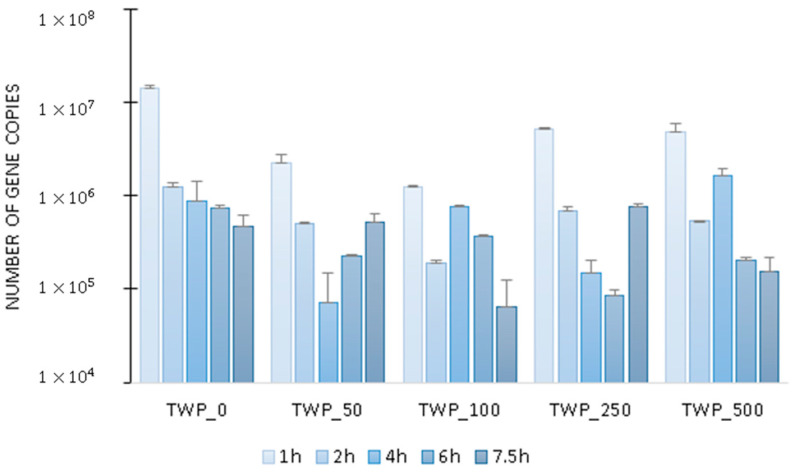
The number of copies of the *nosZ* gene transcripts in the GSBR cycle.

The highest number of *nosZ* gene transcripts characterized initial hours of the working cycle, in which the number of gene copies varied from 14 million (TWP_0) to 1 million (TWP_100) (Figure 5). The average number of transcripts was about 520% higher in the control reactor than in the reactors containing TWPs, indicating that TWPs affect full denitrification in AGS. The reduced efficiency of denitrification in wastewater treatment plants, in turn, is associated with an increase in nitrogen levels in the effluent, which may contribute to eutrophication, characterized by the excessive growth of phytoplankton, including algae and cyanobacteria, leading to a decrease in oxygen levels in the receiver. This results in the deterioration of aquatic ecosystems, reduced biodiversity, and the death of aquatic organisms [47].

**Figure 6:**
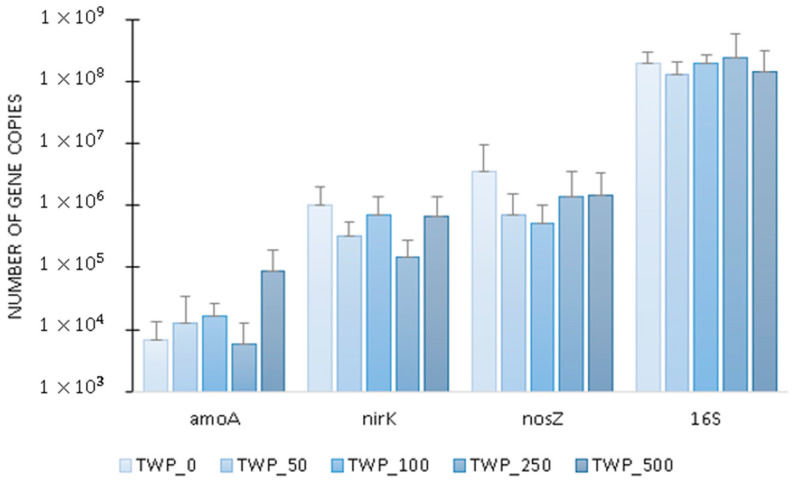
Average copy number of transcripts of the *amoA*, *nirK*, *nosZ* and *16S rRNA* genes in the GSBR cycle.

The average number of *amoA* transcripts increased from 7000 copies in the control group to 86,000 copies in TWP_500 (Figure 6). The copy numbers of the *nirK* and *nosZ* genes in the control averaged 1 million and 4 million, respectively. These values decreased by 33% and 58% in TWP_500, respectively (Figure 6). The average expression of the denitrification genes *nirK* and *nosZ* over the entire cycle of the test reactors was about 410% higher than that of the *amoA* gene (Figure 6). This was due to the higher proportion of heterotrophic denitrifying microorganisms in the biomass compared to autotrophic nitrifying bacteria, resulting from the shorter generation time of the denitrifiers and the advantage associated with the utilization of available organic carbon sources in wastewater [48,49]. The presence of the highest dose of TWPs in the wastewater significantly (r = 0.49) increased the average level of *amoA* transcription in the biomass by about 160% compared to the control reactor. For the denitrification genes, the situation was reversed and their highest activity was recorded in the control reactor where no TWPs were introduced with the wastewater. In the case of the *16S rRNA* gene, no correlation was observed between the average transcript level and the TWP dose in wastewater. Biomass dynamics remained relatively stable; however, a decrease in total activity was noted at the highest TMP dose, which indicated the lowest biomass viability in the wastewater dosed with the 500 mg/L TMP. This may suggest that the overall microbiome activity remained relatively consistent, regardless of the TMP dose, but the metabolism of individual pathways may undergo changes, as illustrated by the data obtained for the other investigated genes. This explains the apparent discrepancy between Figure 2 and Figure 5, which show time specific fluctuations, and Figure 6, which presents mean values summarizing gene expression over the entire cycle. This study was conducted under controlled laboratory conditions, which may not fully reflect the complexity of real wastewater treatment systems. Therefore large-scale field studies, including the use of a mixture of different types of MPs, are necessary to confirm these findings and assess their practical implications in full-scale WWTPs.

## 3. Materials and Methods

### 3.1. Organization of the Experiment

**Figure 7:**
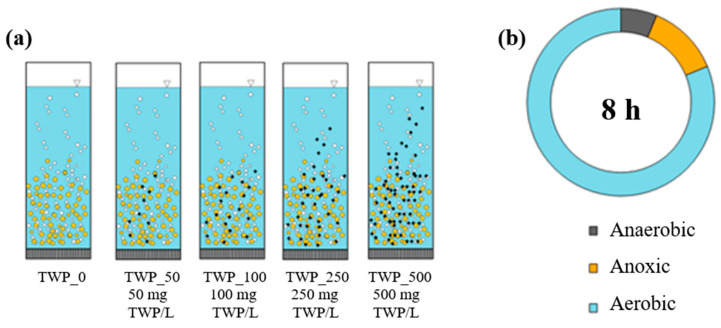
Organization of (**a**) the experiment and (**b**) the GSBR cycle.

In this study, laboratory granular sludge batch reactors (GSBRs) with a working volume of 3 L and a diameter of 10 cm were used. The reactors were operated with programmable controllers at a volumetric exchange rate of 60%/cycle and at 20 °C. Air was supplied (4 L/min) via fine bubble diffusers at the bottom of the reactors. As inoculum, AGS from WWTPs in Lubawa was used. To the reactors, synthetic municipal wastewater [50] was added to avoid uncontrolled contamination with other MPs. Based on the preliminary study, the reactors (TWP_0–TWP_500) were fed with wastewater containing increasing TWP concentrations/L (Figure 7a). To produce TWPs, pristine tires from a leading global manufacturer were milled, and a fraction of 70–200 μm (the most abundant was selected by sieving [51]. An anaerobic/anoxic/aerobic regime applied in the 8 h GSBR cycle is shown in Figure 7b. Each GSBR was operated for more than 300 cycles. The mixed liquor suspended solids in the GSBRs was maintained at about 6 g MLSS/L (grams of mixed liquor suspended solids per liter).

### 3.2. Technological Analyses

Measurements of pollutant removal kinetics and molecular samplings were performed in the stable operation of the reactor in which the efficiency of chemical oxygen demand (COD), total nitrogen (TN) and total phosphorus (TP) removal exceeded 90%. Kinetic studies were performed in duplicate in all GSBRs and included the measurement of ammonium, nitrites and nitrates (cuvette tests, Hach Lange, Düsseldorf, Germany). The AGS concentration in the GSBRs (g MLSS/L, g MLVSS/L (grams of mixed liquor volatile suspended solids per liter)) was analyzed according to APHA 2012 [52].

### 3.3. Molecular Analyses

The aim of the study was to quantify the total microbiome (*16S rRNA*), ammonia monooxygenase (*amoA* gene), nitrite reductase (*nirK* gene) and nitric oxide reductase (*nosZ* gene) number of transcripts of genes coding during the operation cycle of GSBR under conditions of varying TWP concentrations in wastewater. In the study, the use of the *nirS* gene was abandoned due to the wider distribution of the *nirK* gene among bacteria in AGS [53]. Granular sludge samples were collected from all five reactors at 1 h, 2 h, 4 h, 6 h and 7.5 h of the GSBR cycle, fixed in phenozol (A&A Biotechnology, Gdańsk, Poland) and stored at −80 °C until analysis. RNA isolation was performed from 0.5 mL of the fixed pellet using the Total RNA Mini kit (A&A Biotechnology) according to the manufacturer’s instructions. DNAse (A&A Biotechnology) was added to the isolated RNA to remove DNA contamination. The quality and quantity of the obtained RNA were examined in a NanoDrop Lite spectrophotometer (Thermo Scientific, Waltham, MA, USA). Equal amounts of RNA (500 µg) from each isolation were used as templates for cDNA synthesis using the RevertAid™ H Minus First Strand cDNA Synthesis Kit (Fermentas, Waltham, MA, USA). Plasmids containing the cloned genes that were selected for the study were used to obtain standard curves [54]. The Clone Jet PCR Cloning Kit (Thermo Scientific) and chemically competent cells of *Escherichia coli* strain JM109 (Promega, Madison, WI, USA) were used for cloning in addition to a standard curve for *16S rRNA,* which was constructed using isolated genomic DNA from *Escherichia coli* strain JM109 (Promega).

Real-time PCR for each sample was performed in triplicate according to the thermal profiles described in the Table A1. The reaction mixture consisted of 10 µL of Power SYBR Green PCR Master Mix (Applied Biosystems, Foster City, CA, USA), primers (100 nM for *16S rRNA* and *amoA*, 150 nM for *nirK* and 200 nM for *nosZ*), 0.5 µL of cDNA and water to final volume of 20 µL. Gene amplifications were performed using the 7500 Real-Time PCR System (Applied Biosystems). The specificity of the amplification reaction was confirmed by dissociation curves and agarose electrophoresis in the presence of a molecular marker (DNA Marker 1, A&A Biotechnology).

### 3.4. Statistical Analysis

Calculations and statistical analysis were performed using Statistica 13.0 software. The copy number of gene transcripts was correlated with TWP dose using Pearson correlation analysis (*p* < 0.05).

## 4. Conclusions

The present study shows that the presence of TWPs affects the kinetics of pollutant transformation in GSBR: it increases the efficiency of ammonium–nitrogen removal from wastewater and accelerates the production of oxidized nitrogen forms by the biomass.

The presence of rubber and aromatic substrates leached from it promotes increases ammonium nitrification, while negatively affecting the activity of genes related to the denitrification cycle. The activity of nitrite reductase was gradually inhibited with an increasing TWP dose in wastewater, while the activity of nitric oxide reductase decreased significantly even at the lowest TWP dose. This disparity may be attributed to differences in the sensitivity of nitrite reductase and nitric oxide reductase to oxidative stress, toxic compounds leached from TWPs, or alterations in redox conditions within the granular sludge. Additionally, TMP does not alter the overall activity of the community, but it clearly affects specific metabolic pathways related to nitrogen metabolism. This is a disturbing phenomenon, suggesting that complete denitrification may be inhibited and nitrogen oxide emissions from wastewater treatment plants may increase in the presence of TWPs in wastewater. The results highlight the need for further research on mitigation strategies to reduce the impact of TWPs in WWTPs. Possible approaches include reducing the impact of TMP through its removal during the mechanical treatment process, e.g., by coagulation.

## Data Availability

The data presented in this study are available on request from the corresponding author.

## Appendix A

**Table A1:** Primers and conditions applied in real-time PCR.

Gene	Primer Set	Thermal Profile	Reference
*16S rRNA*	519F/907R	94 °C/15 s 50 °C/45 s 60 °C/45 s	Lane (1991) [55]
*amoA*	amoA1F/amoA2R	94 °C/15 s 50 °C/45 s 60 °C/45 s	Rotthauwe et al. (1997) [56]
*nirK*	F1aCu/R3Cu	94 °C/15 s 55 °C/1 min 60 °C/1 min	Throbäck et al. (2004) [57]
*nosZ*	NosZ-F/NosZ1622R	94 °C/15 s 60 °C/2.5 min	Throbäck et al. (2004) [57]

**Table A2:** Nitrogen compound transformations in GSBRs.

TWP Concentration mg/L	N-NH_4_ mg/(L·h)	N-Nox mg/(L·h)
0	3.9	2.0
50	4.1	2.3
100	3.7	1.4
250	4.5	2.5
500	4.5	3.2

**Table A3:** Pearson correlation.

	Dose	Time	*amoA*	*nirK*	*nosZ*	*16S rRNA*
Dose	1.000000	0.000000	0.489366	−0.095148	−0.077729	−0.039697
Time	0.000000	1.000000	0.047333	−0.609123	−0.472511	−0.121096
*amoA*	0.489366	0.047333	1.000000	−0.104749	0.042104	−0.179776
*nirK*	−0.095148	−0.609123	−0.104749	1.000000	0.739879	0.221573
*nosZ*	−0.077729	−0.472511	0.042104	0.739879	1.000000	0.220938
*16S rRNA*	−0.039697	−0.121096	−0.179776	0.221573	0.220938	1.000000
